# Supplementary figures and images for: Presence of microplastics and microparticles in Oregon Black Rockfish sampled near marine reserve areas
Source: PeerJ. 2023 Feb 14;11:e14564. doi: 10.7717/peerj.14564 (PMC9936869; doi:10.7717/peerj.14564)

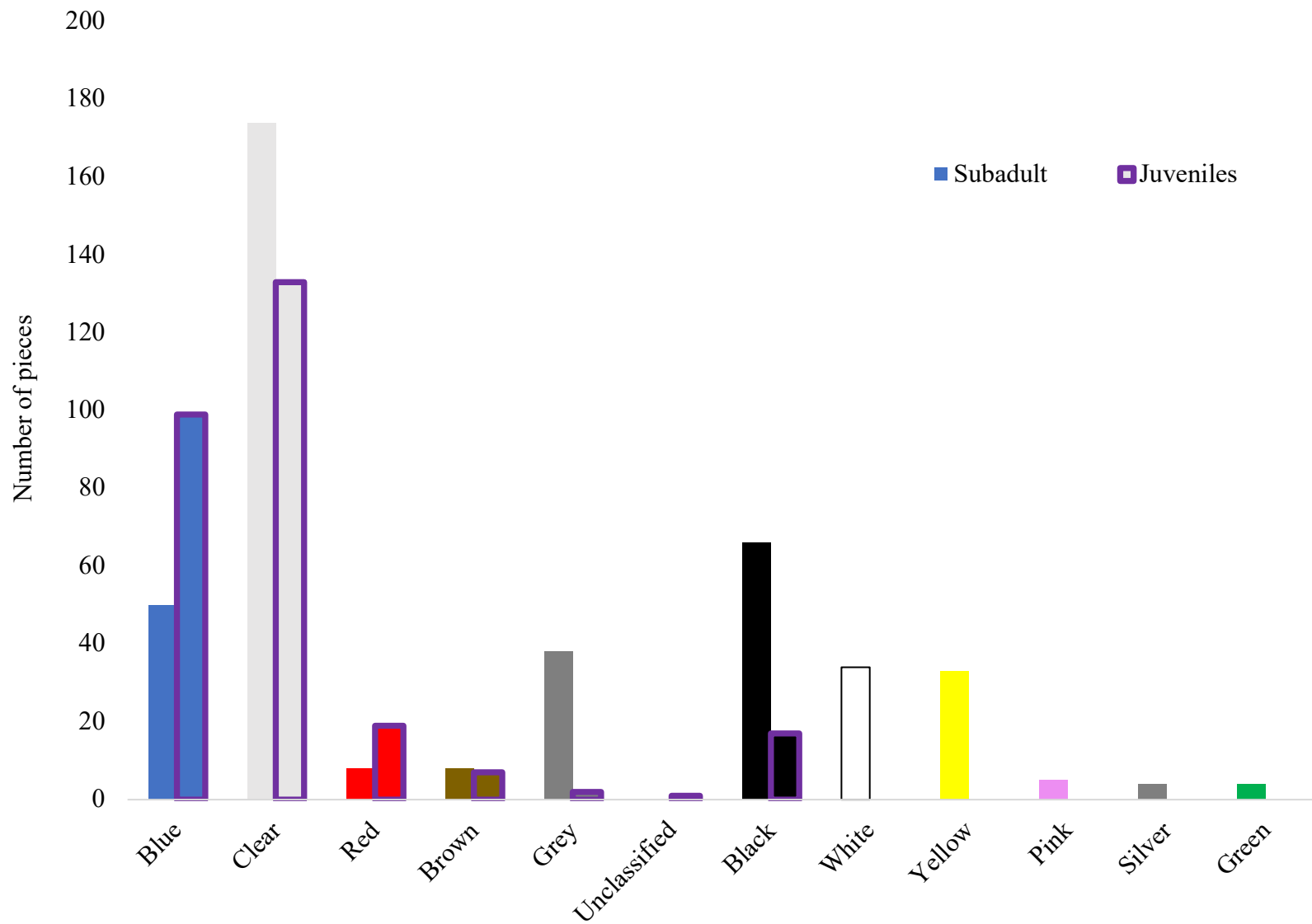

Supplement: Supplemental Information 2 [file peerj-11-14564-s002.pdf]

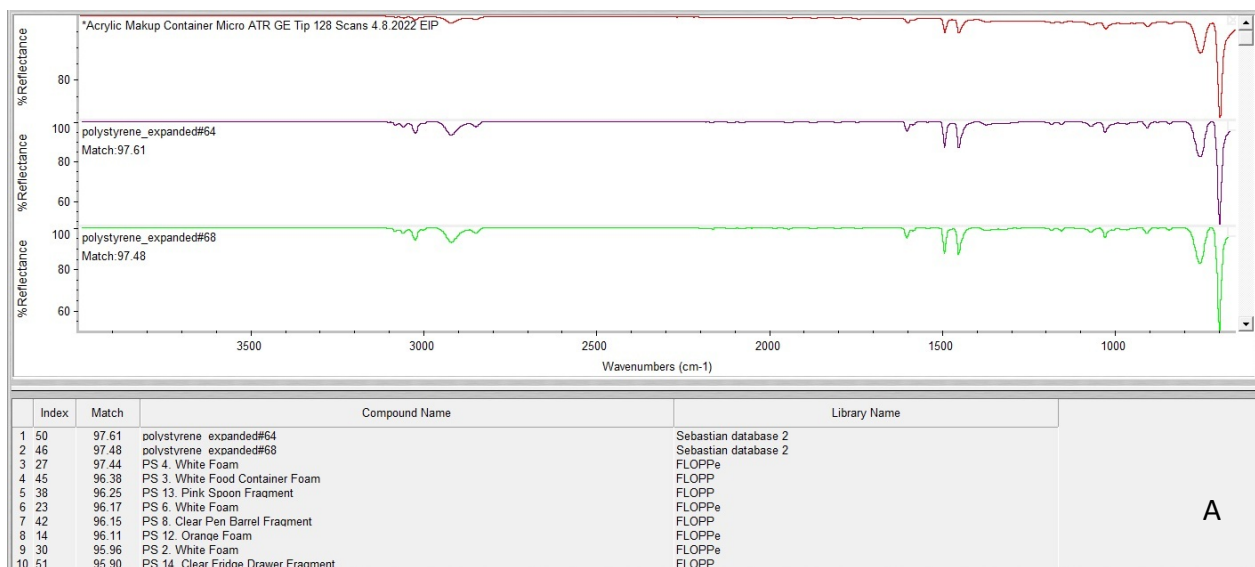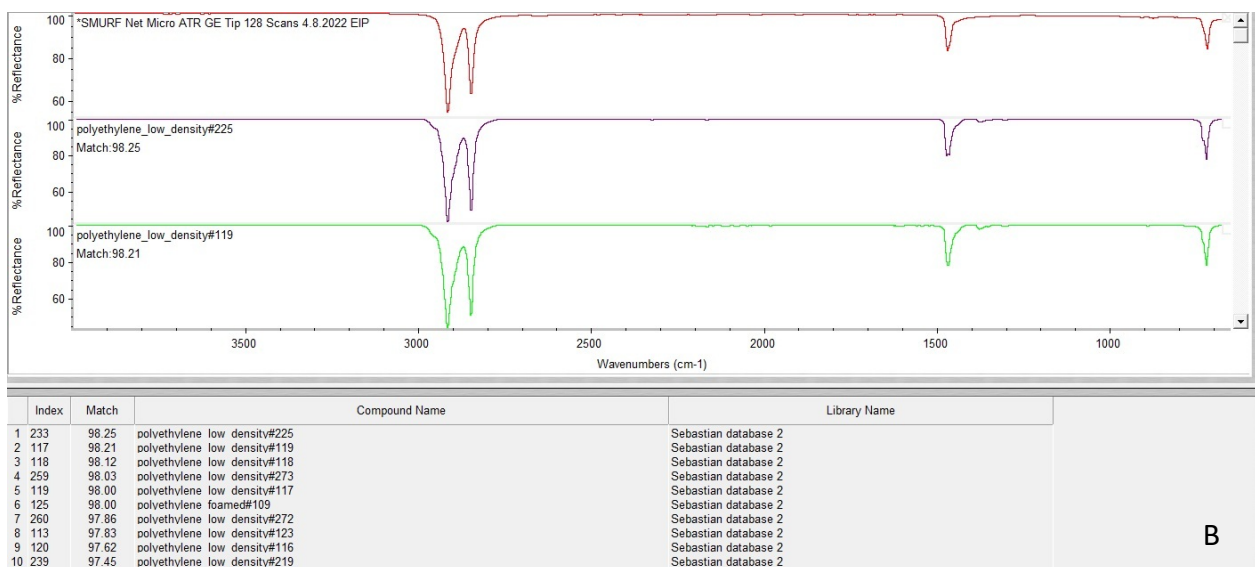

Supplement: Supplemental Information 3 [file peerj-11-14564-s003.pdf]
