# Supplementary material for: Presence of microplastics and microparticles in Oregon Black Rockfish sampled near marine reserve areas
Source: PeerJ. 2023 Feb 14;11:e14564. doi: 10.7717/peerj.14564 (PMC9936869; doi:10.7717/peerj.14564)
Supplement: Supplemental Information 4 [file peerj-11-14564-s004.docx]

library(car)

Error: package or namespace load failed for ‘car’ in library.dynam(lib, package, package.lib):

DLL ‘stringi’ not found: maybe not installed for this architecture?

> library(carData)

> library(CARS)

> library(ggplot2)

> library(multcomp)

> library(cowplot)

>

> subadult_code$Loc <- (subadult_code$'Location 2.0')

> subadult_code$Loc

[1] "Newport" "Newport" "Newport" "Newport" "Newport" "Newport"

[7] "Newport" "Newport" "Newport" "Newport" "Newport" "Newport"

[13] "Newport" "Newport" "Cascade Head" "Cascade Head" "Cascade Head" "Cascade Head"

[19] "Cascade Head" "Cascade Head" "Cascade Head" "Cascade Head" "Cascade Head" "Cascade Head"

[25] "Cape Perpetua" "Cape Perpetua" "Cape Perpetua" "Cape Perpetua" "Cape Perpetua" "Cape Perpetua"

[31] "Cape Perpetua" "Cape Perpetua" "Cape Perpetua" "Cape Perpetua" "Cape Perpetua" "Cape Perpetua"

[37] "Cape Perpetua" "Cape Falcon" "Cape Falcon" "Cape Falcon" "Cape Falcon" "Cape Falcon"

[43] "Cape Falcon" "Cape Falcon" "Cape Falcon" "Cape Falcon" "Cape Falcon" "Redfish Rocks"

[49] "Redfish Rocks" "Redfish Rocks" "Redfish Rocks" "Redfish Rocks" "Redfish Rocks" "Redfish Rocks"

[55] "Redfish Rocks" "Redfish Rocks" "Redfish Rocks" "Redfish Rocks"

> LO <-factor (subadult_code$Loc, levels = c("Cape Falcon", "Cascade Head", "Newport","Cape Perpetua","Redfish Rocks"))

>

>

>

> juvenile_code$Loc2 <- (juvenile_code$`Location 2.0`)

>

>

> ina1 = as.factor(subadult_code$Loc)

> fit1<-glm(subadult_code$Pieces ~ ina1, data = subadult_code, family = poisson)

> infct1 = mcp(ina1 = 'Tukey')

> infct1

$ina1

[1] "Tukey"

attr(,"interaction_average")

[1] FALSE

attr(,"covariate_average")

[1] FALSE

attr(,"class")

[1] "mcp"

> summary(glht(fit1, linfct = mcp (ina1= "Tukey")))

Simultaneous Tests for General Linear Hypotheses

Multiple Comparisons of Means: Tukey Contrasts

Fit: glm(formula = subadult_code$Pieces ~ ina1, family = poisson,

data = subadult_code)

Linear Hypotheses:

Estimate Std. Error z value Pr(>|z|)

Cape Perpetua - Cape Falcon == 0 -0.76232 0.14734 -5.174 < 0.001 ***

Cascade Head - Cape Falcon == 0 -0.25014 0.13683 -1.828 0.35470

Newport - Cape Falcon == 0 -0.79669 0.14555 -5.474 < 0.001 ***

Redfish Rocks - Cape Falcon == 0 -0.87398 0.16141 -5.415 < 0.001 ***

Cascade Head - Cape Perpetua == 0 0.51218 0.15505 3.303 0.00842 **

Newport - Cape Perpetua == 0 -0.03437 0.16279 -0.211 0.99955

Redfish Rocks - Cape Perpetua == 0 -0.11166 0.17712 -0.630 0.96987

Newport - Cascade Head == 0 -0.54654 0.15334 -3.564 0.00320 **

Redfish Rocks - Cascade Head == 0 -0.62384 0.16847 -3.703 0.00206 **

Redfish Rocks - Newport == 0 -0.07729 0.17562 -0.440 0.99214

---

Signif. codes: 0 ‘***’ 0.001 ‘**’ 0.01 ‘*’ 0.05 ‘.’ 0.1 ‘ ’ 1

(Adjusted p values reported -- single-step method)

> CISub <- summary(glht(fit1, linfct = mcp (ina1= "Tukey")))

> confint(CISub)

Simultaneous Confidence Intervals

Multiple Comparisons of Means: Tukey Contrasts

Fit: glm(formula = subadult_code$Pieces ~ ina1, family = poisson,

data = subadult_code)

Quantile = 2.7239

95% family-wise confidence level

Linear Hypotheses:

Estimate lwr upr

Cape Perpetua - Cape Falcon == 0 -0.76232 -1.16367 -0.36097

Cascade Head - Cape Falcon == 0 -0.25014 -0.62285 0.12256

Newport - Cape Falcon == 0 -0.79669 -1.19314 -0.40024

Redfish Rocks - Cape Falcon == 0 -0.87398 -1.31365 -0.43431

Cascade Head - Cape Perpetua == 0 0.51218 0.08985 0.93450

Newport - Cape Perpetua == 0 -0.03437 -0.47779 0.40905

Redfish Rocks - Cape Perpetua == 0 -0.11166 -0.59411 0.37079

Newport - Cascade Head == 0 -0.54654 -0.96422 -0.12887

Redfish Rocks - Cascade Head == 0 -0.62384 -1.08273 -0.16494

Redfish Rocks - Newport == 0 -0.07729 -0.55567 0.40109

>

> summary(fit1)

Call:

glm(formula = subadult_code$Pieces ~ ina1, family = poisson,

data = subadult_code)

Deviance Residuals:

Min 1Q Median 3Q Max

-4.3589 -1.7714 -0.6726 0.6134 7.7366

Coefficients:

Estimate Std. Error z value Pr(>|z|)

(Intercept) 2.50144 0.09054 27.629 < 2e-16 ***

ina1Cape Perpetua -0.76232 0.14734 -5.174 2.29e-07 ***

ina1Cascade Head -0.25014 0.13683 -1.828 0.0675 .

ina1Newport -0.79669 0.14555 -5.474 4.41e-08 ***

ina1Redfish Rocks -0.87398 0.16141 -5.415 6.14e-08 ***

---

Signif. codes: 0 ‘***’ 0.001 ‘**’ 0.01 ‘*’ 0.05 ‘.’ 0.1 ‘ ’ 1

(Dispersion parameter for poisson family taken to be 1)

Null deviance: 351.64 on 57 degrees of freedom

Residual deviance: 298.26 on 53 degrees of freedom

AIC: 501.69

Number of Fisher Scoring iterations: 5

>

> ina2 = as.factor(juvenile_code$Loc2)

> fit2<-glm(juvenile_code$Pieces ~ ina2, data = juvenile_code, family = poisson)

> infct2 = mcp(ina2 = 'Tukey')

> summary(glht(fit2, linfct = mcp (ina2= "Tukey")))

Simultaneous Tests for General Linear Hypotheses

Multiple Comparisons of Means: Tukey Contrasts

Fit: glm(formula = juvenile_code$Pieces ~ ina2, family = poisson,

data = juvenile_code)

Linear Hypotheses:

Estimate Std. Error z value Pr(>|z|)

Otter Rock - Cape Foulweather == 0 0.31488 0.17285 1.822 0.145

Unknown - Cape Foulweather == 0 -0.08004 0.59914 -0.134 0.989

Unknown - Otter Rock == 0 -0.39493 0.58101 -0.680 0.759

(Adjusted p values reported -- single-step method)

> CIjuv <- summary(glht(fit2, linfct = mcp (ina2= "Tukey")))

> confint(CIjuv)

Simultaneous Confidence Intervals

Multiple Comparisons of Means: Tukey Contrasts

Fit: glm(formula = juvenile_code$Pieces ~ ina2, family = poisson,

data = juvenile_code)

Quantile = 2.2848

95% family-wise confidence level

Linear Hypotheses:

Estimate lwr upr

Otter Rock - Cape Foulweather == 0 0.31488 -0.08006 0.70983

Unknown - Cape Foulweather == 0 -0.08004 -1.44899 1.28890

Unknown - Otter Rock == 0 -0.39493 -1.72244 0.93258

>

> summary(fit2)

Call:

glm(formula = juvenile_code$Pieces ~ ina2, family = poisson,

data = juvenile_code)

Deviance Residuals:

Min 1Q Median 3Q Max

-2.9842 -1.8110 -0.2184 0.8481 4.2232

Coefficients:

Estimate Std. Error z value Pr(>|z|)

(Intercept) 1.17865 0.16013 7.361 1.83e-13 ***

ina2Otter Rock 0.31488 0.17285 1.822 0.0685 .

ina2Unknown -0.08004 0.59914 -0.134 0.8937

---

Signif. codes: 0 ‘***’ 0.001 ‘**’ 0.01 ‘*’ 0.05 ‘.’ 0.1 ‘ ’ 1

(Dispersion parameter for poisson family taken to be 1)

Null deviance: 176.37 on 65 degrees of freedom

Residual deviance: 172.40 on 63 degrees of freedom

AIC: 371.71

Number of Fisher Scoring iterations: 5

>

>

> subadult <- ggplot(subadult_code, aes(x=LO, y=Pieces,fill=LO)) +

+ geom_boxplot(outlier.color="grey", alpha = 1.2, outlier.size = 10) +

+ scale_fill_manual(values = c("blue", "pink", "cornsilk", "grey", "coral1"))+

+ labs(x= "Location", y= "Pieces per fish") +

+ theme_classic() +

+ theme(axis.text.x = element_text(color = "black", size = 50),

+ axis.text.y = element_text(color = "black", size = 50),

+ axis.title.x = element_text(color = "black", size = 70),

+ axis.title.y = element_text(color = "black", size = 70)) +

+ stat_summary(fun=mean, geom="point", shape=20, size=20, color="black", fill="black")+ # this pts means on graph

+ theme(legend.position="none")

>

> subadult2 <- subadult + geom_hline(yintercept=5, linetype="dashed", color = "purple", size = 8)

>

> plot2 <-subadult2

> plot(plot2)

>

>

>

> juvenile <- ggplot(juvenile_code, aes(x=Loc2, y=Pieces, fill=Loc2)) +

+ geom_boxplot(outlier.color="grey" , alpha =1.2, outlier.size = 10) +

+ labs(x= "Location", y= "Pieces per fish") +

+ theme_classic() +

+ theme(axis.text.x = element_text(color = "black", size = 50),

+ axis.text.y = element_text(color = "black", size = 50),

+ axis.title.x = element_text(color = "black", size = 70),

+ axis.title.y = element_text(color = "black", size = 70)) +

+ stat_summary(fun=mean, geom="point", shape=20, size=20, color="black", fill="white")+ # this pts means on graph

+ theme(legend.position="none")

>

> juvenile2 <-juvenile + geom_hline(yintercept=1.82, linetype="dashed", color = "purple", size = 8)

> plot3 <- (juvenile2 + scale_fill_grey())

> plot (plot3)

>

>

>

> plot_grid(plot2, plot3, labels = c("A", "B"), ncol = 1, nrow =2)

> subadult <- ggplot(subadult_code, aes(x=LO, y=Pieces,fill=LO)) +

+ geom_boxplot(outlier.color="grey", alpha = 1.2, outlier.size = 10) +

+ scale_fill_manual(values = c("blue", "pink", "cornsilk", "grey", "coral1"))+

+ labs(x= "Location", y= "Pieces per fish") +

+ theme_classic() +

+ theme(axis.text.x = element_text(color = "black", size = 40),

+ axis.text.y = element_text(color = "black", size = 40),

+ axis.title.x = element_text(color = "black", size = 70),

+ axis.title.y = element_text(color = "black", size = 70)) +

+ stat_summary(fun=mean, geom="point", shape=20, size=20, color="black", fill="black")+ # this pts means on graph

+ theme(legend.position="none")

>

> subadult2 <- subadult + geom_hline(yintercept=5, linetype="dashed", color = "purple", size = 8)

>

> plot2 <-subadult2

> plot(plot2)

>

>

>

> juvenile <- ggplot(juvenile_code, aes(x=Loc2, y=Pieces, fill=Loc2)) +

+ geom_boxplot(outlier.color="grey" , alpha =1.2, outlier.size = 10) +

+ labs(x= "Location", y= "Pieces per fish") +

+ theme_classic() +

+ theme(axis.text.x = element_text(color = "black", size = 40),

+ axis.text.y = element_text(color = "black", size = 40),

+ axis.title.x = element_text(color = "black", size = 70),

+ axis.title.y = element_text(color = "black", size = 70)) +

+ stat_summary(fun=mean, geom="point", shape=20, size=20, color="black", fill="white")+ # this pts means on graph

+ theme(legend.position="none")

>

> juvenile2 <-juvenile + geom_hline(yintercept=1.82, linetype="dashed", color = "purple", size = 8)

> plot3 <- (juvenile2 + scale_fill_grey())

> plot (plot3)

>

>

>

> plot_grid(plot2, plot3, labels = c("A", "B", size =40), ncol = 1, nrow =2)

> subadult <- ggplot(subadult_code, aes(x=LO, y=Pieces,fill=LO)) +

+ geom_boxplot(outlier.color="grey", alpha = 1.2, outlier.size = 10) +

+ scale_fill_manual(values = c("blue", "pink", "cornsilk", "grey", "coral1"))+

+ labs(x= "Location", y= "Pieces per fish") +

+ theme_classic() +

+ theme(axis.text.x = element_text(color = "black", size = 40),

+ axis.text.y = element_text(color = "black", size = 40),

+ axis.title.x = element_text(color = "black", size = 70),

+ axis.title.y = element_text(color = "black", size = 70)) +

+ stat_summary(fun=mean, geom="point", shape=20, size=20, color="black", fill="black")+ # this pts means on graph

+ theme(legend.position="none")

>

> subadult2 <- subadult + geom_hline(yintercept=5, linetype="dashed", color = "purple", size = 8)

>

> plot2 <-subadult2

> plot(plot2)

>

>

>

> juvenile <- ggplot(juvenile_code, aes(x=Loc2, y=Pieces, fill=Loc2)) +

+ geom_boxplot(outlier.color="grey" , alpha =1.2, outlier.size = 10) +

+ labs(x= "Location", y= "Pieces per fish") +

+ theme_classic() +

+ theme(axis.text.x = element_text(color = "black", size = 40),

+ axis.text.y = element_text(color = "black", size = 40),

+ axis.title.x = element_text(color = "black", size = 70),

+ axis.title.y = element_text(color = "black", size = 70)) +

+ stat_summary(fun=mean, geom="point", shape=20, size=20, color="black", fill="white")+ # this pts means on graph

+ theme(legend.position="none")

>

> juvenile2 <-juvenile + geom_hline(yintercept=1.82, linetype="dashed", color = "purple", size = 8)

> plot3 <- (juvenile2 + scale_fill_grey())

> plot (plot3)

>

>

>

> plot_grid(plot2, plot3, labels = c("A", "B"), label_size = 40, ncol = 1, nrow =2)

> subadult <- ggplot(subadult_code, aes(x=LO, y=Pieces,fill=LO)) +

+ geom_boxplot(outlier.color="grey", alpha = 1.2, outlier.size = 10) +

+ scale_fill_manual(values = c("blue", "pink", "cornsilk", "grey", "coral1"))+

+ labs(x= "Location", y= "Pieces per fish") +

+ theme_classic() +

+ theme(axis.text.x = element_text(color = "black", size = 40),

+ axis.text.y = element_text(color = "black", size = 40),

+ axis.title.x = element_text(color = "black", size = 70),

+ axis.title.y = element_text(color = "black", size = 70)) +

+ stat_summary(fun=mean, geom="point", shape=20, size=20, color="black", fill="black")+ # this pts means on graph

+ theme(legend.position="none")

>

> subadult2 <- subadult + geom_hline(yintercept=5, linetype="dashed", color = "purple", size = 8)

>

> plot2 <-subadult2

> plot(plot2)

>

>

>

> juvenile <- ggplot(juvenile_code, aes(x=Loc2, y=Pieces, fill=Loc2)) +

+ geom_boxplot(outlier.color="grey" , alpha =1.2, outlier.size = 10) +

+ labs(x= "Location", y= "Pieces per fish") +

+ theme_classic() +

+ theme(axis.text.x = element_text(color = "black", size = 40),

+ axis.text.y = element_text(color = "black", size = 40),

+ axis.title.x = element_text(color = "black", size = 70),

+ axis.title.y = element_text(color = "black", size = 70)) +

+ stat_summary(fun=mean, geom="point", shape=20, size=20, color="black", fill="white")+ # this pts means on graph

+ theme(legend.position="none")

>

> juvenile2 <-juvenile + geom_hline(yintercept=1.82, linetype="dashed", color = "purple", size = 8)

> plot3 <- (juvenile2 + scale_fill_grey())

> plot (plot3)

>

>

>

> plot_grid(plot2, plot3, labels = c("A", "B"), label_size = 70, ncol = 1, nrow =2)
